# Vision System-Based Design and Assessment of a Novel Shoulder Joint Mechanism for an Enhanced Workspace Upper Limb Exoskeleton

**DOI:** 10.1155/2018/6019381

**Published:** 2018-06-03

**Authors:** Eduardo Piña-Martínez, Ricardo Roberts, Salvador Leal-Merlo, Ernesto Rodriguez-Leal

**Affiliations:** ^1^Escuela de Ingeniería y Ciencias, Ave. Eugenio Garza Sada 2501, Tecnologico de Monterrey, 64849 Monterrey, NL, Mexico; ^2^Massachusetts Institute of Technology, 77 Massachusetts Ave., Cambridge, MA 02139, USA

## Abstract

Exoskeletons arise as the common ground between robotics and biomechanics, where rehabilitation is the main field in which these two disciplines find cohesion. One of the most relevant challenges in upper limb exoskeleton design relies in the high complexity of the human shoulder, where current devices implement elaborate systems only to emulate the drifting center of rotation of the shoulder joint. This paper proposes the use of 3D scanning vision technologies to ease the design process and its implementation on a variety of subjects, while a motion tracking system based on vision technologies is applied to assess the exoskeleton reachable workspace compared with an asymptomatic subject. Furthermore, the anatomic fitting index is proposed, which compares the anatomic workspace of the user with the exoskeleton workspace and provides insight into its features. This work proposes an exoskeleton architecture that considers the clavicle motion over the coronal plane whose workspace is determined by substituting the direct kinematics model with the dimensional parameters of the user. Simulations and numerical examples are used to validate the analytical results and to conciliate the experimental results provided by the vision tracking system.

## 1. Introduction

Automated manufacturing has boosted productivity and improved the workers' quality of life in the past decades. However, industrial robots have remained isolated in factories in order to avoid unintended harm to nearby individuals or facilities due to the lack of advanced awareness on its surroundings. Robotic advancements have closed this gap by adding safety features and improving robot sensory awareness. Simultaneously, younger generations have grown habituated with computing devices and therefore interact more readily with new devices and digital technologies. Exoskeletons represent the epitome of these two trends and the final blending between human and machine, a mechanism whose joints and links correspond to those of the human body and mimic the user movements [1].

Originally, exoskeletons were based on industrial robot architectures, adopting similar actuators, mechanisms, and materials [2]. This approach, however, is suboptimal for exoskeletons that try to mimic the human body mobility. Joint complexity and the dimensional variability between individuals limit the effectiveness of industrial robot architectures, and therefore, new exoskeleton designs have been proposed in the past years to tackle these challenges [3–5].

Robotic exoskeletons comprise lower limb and upper limb devices that act directly over the corresponding part of the human body [6]. Each class of exoskeletons encompasses its own design needs, such as high-torque motors or greater range of motion for lower and upper limbs, respectively [3, 5, 7, 8]. A thorough analysis of lower limb exoskeleton developments is presented by Dollar and Herr in [9], where the authors focus on advances in control and challenges in actuation and propose the concept of metabolic cost as a tool to measure the device effectiveness. The work also discusses exoskeleton applications such as a force enhancer for healthy individuals or an assistive tool for the physically challenged. Complementarily, an extensive analysis of upper limb exoskeletons for rehabilitation is presented by Lo and Xie [10], where the authors emphasize the importance of the proper mimic of the clavicle for an adequate shoulder mobility. Furthermore, the paper presents current advances in actuation and control technology, analyzing different approaches for receiving and sending information to the user through EMG and haptic feedback, respectively.

The abovementioned research papers show that exoskeletons provide motion with mechanisms that are attached to the body and move parallel to their corresponding biological components. This wearable configuration requires further analysis of the human anatomy but enables complete control of all the limb parts independently [10]. It is well known that limb posture control is straightforward for the elbow due to its single rotational degree of freedom (henceforth denoted as DoF). For the shoulder, however, limb posture control represents a challenging task as this structure includes the glenohumeral, acromioclavicular, sternoclavicular, and scapulothoracic joints. The development of a device that accurately mimics all these joints behavior will represent an advancement in exoskeleton technology [11].

Elbow joint exoskeletons have been developed using novel control algorithms or actuating mechanisms. For example, a single DoF robotic exoskeleton for motion assist of the elbow joint was developed by Kiguchi et al. [12], controlling its angular position and mechanical impedance by reading and characterizing electromyographic signals of the user. In addition, it is possible to find in the literature that different work on actuation has been performed for its application on this particular joint: Zhang et al. [13] present the development of a single DoF exoskeletal joint for the elbow that is based on curved pneumatic muscle actuators, while Miranda et al. [14] propose and analyze a linear actuator in order to obtain similar torque constraints as they appear in the human biceps. Furthermore, wrist exoskeletal joints have been developed and studied for its use in rehabilitation therapy [15].

The elbow complexity pales in comparison to the shoulder, which requires a thorough analysis to understand its mobility. Accordingly, exoskeletons depend on at least four DoF to replicate this joint behavior [11]. Complexity arises from the interactions between the clavicle, scapula, and humerus bones, which create a drifting center of rotation (CR). Upper arm exoskeletons should follow this CR in order to minimize ill effects [11]. Nevertheless, some exoskeletons present spherical joints as shoulders [1, 16], which simplifies mechanical complexity due to its fixed CR but limits the user range of motion. This analysis is further explored with motion tracking technology [17], where daily task movements are analyzed in order to construct an appropriate kinematic model of the upper limb. The work presented in this paper combines state-of-the-art motion tracking techniques that were then used by Piña-Martínez and Rodriguez-Leal [18] to analyze the knee mobility with novel kinematic models. This allows to characterize the mobility of the human joints as seen from an external structure (e.g., exoskeleton), which eases the subsequent task of biomimetic design for the development of exoskeletal mechanisms, and can be used for joints with complex CR behavior (e.g., the shoulder).

Shoulder CR mobility represents a topic of interest in upper limb exoskeleton design, for applications that require optimal motion tracking [19, 20] that considers an ambulatory CR, where at least one DoF in the clavicle area supplementary to the three abovementioned shoulder DoF is required [21]. This additional DoF has been implemented as a passive prismatic joint in a mechanism with three actuated joints driven by motors attached to a chair by cables [22]. Although this design implements a mobile CR, its cable-driven actuation and bulky sliding mechanism are not suitable for a portable device. Other developments on upper limb exoskeletons that consider the CR mobility are focused on the scapula [23], whose mobility is mainly led and constrained by the clavicle; however, little to no assessment methodologies had been developed in order to evaluate these exoskeletal architectures.

Upper body exoskeletons with variant CR have also been patented. For example, an upper limb design implements a parallel robot with seven linkages that drive a platform over the shoulder, where six links are attached to the user's torso and the remaining link connects the platform to the arm [24]. This configuration enables mobility assistance to the arm, but further work is required to implement such concept in a device that exerts the necessary forces and protects the user from unsafe postures.

Challenges in exoskeleton design include posture determination and motion control of its multiple DoF robotic components [10]. Furthermore, the highly complex mechanics and redundancy of the human joint structures represent a current object of study [25, 26]. These two fields have been studied separately by the robotic and medical communities for several decades, but have recently found common ground in the field of wearable robotics. One key application of this nascent discipline includes rehabilitation, where poststroke robot-assisted therapy has improved patient recovery outcomes [27]. These results encourage further development of upper limb exoskeletons for rehabilitation and motion assistance.

This paper extends the work presented in [18] for the shoulder joint, hereby is presented a novel design and assessment method for the mechanical architecture of upper limb exoskeletons. The paper is organized as follows: Section 2 includes a motion study of the human arm, determines its skeletal structure, and proposes a method for its comparison with exoskeletal structures.  Section 3 presents the design process of a novel exoskeletal mechanism and develops the kinematic analysis of the exoskeleton based on anatomical measurements.  Section 4 presents the experimental setup for the data acquisition of the anatomical workspace by using state-of-the-art motion capture technology, as well as the analytical workspace acquisition by the use of the exoskeleton mathematical model.  Section 5 discusses the prototype and validates the theoretical workspace with experimental data. Finally, the paper presents conclusions and suggestions for further work in Section 6.

## 2. Arm Motion and Anatomic Fitting Index

**Figure 1:**
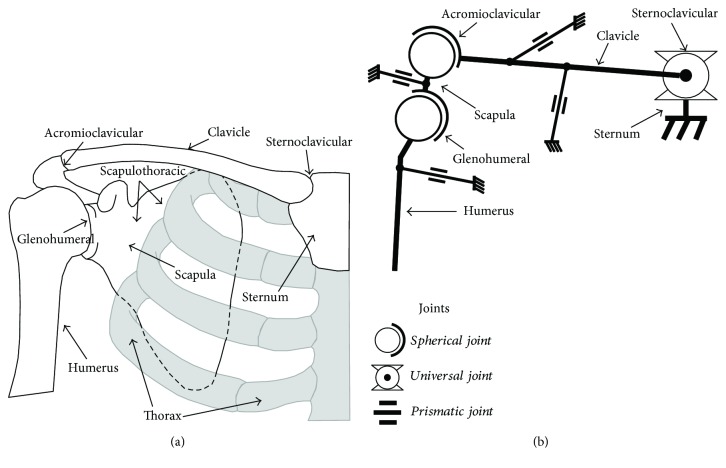
(a) Human shoulder skeletal system and (b) equivalent kinematic model of the shoulder complex with their corresponding joints.

The shoulder complex includes the clavicle, humerus, and scapula bones and its mechanical interactions extend to the thorax, which supports the system weight.  Figure 1(a) shows a frontal view of the shoulder complex and its four joints: glenohumeral, acromioclavicular, sternoclavicular, and scapulothoracic. The thorax and scapula interact physically, but the scapulothoracic articulation cannot be considered an anatomical joint due to the lack of connective tissue between these two structures [11, 28]. The scapula and humerus compose the glenohumeral joint, which is commonly considered the shoulder joint and is usually modeled as a ball and socket mechanism due to the semispherical surface of the humeral head. Furthermore, the acromioclavicular and sternoclavicular joints connect the clavicle to the scapula and thorax, respectively, and enable the complex shoulder behavior analyzed in this work [11]. The acromioclavicular articulation is considered a synovial joint that shows a gliding motion; however, due to the marginal gliding contribution given by its size, this articulation is commonly considered as a noncanonical spherical joint [1, 3, 23, 25], which can be represented as three rotational joints whose nonorthogonal axes intersect a point in space. Thus, the sternoclavicular, acromioclavicular, and glenohumeral articulations are modeled in this work as universal, spherical, and spherical joints, respectively. These joints construct an overactuated mechanism due to the redundant actuation induced by muscles that interact with the shoulder complex. Furthermore, this system includes multiple constraints exerted by the complex interaction of tendons and ligaments that limit the system motion. Note that both constraints and actuators are modeled as prismatic joints in Figure 1(b). Also, the resulting mechanism presents redundancy due to the interaction of two spherical joints that are connected in series (glenohumeral and sternoclavicular).

**Figure 2:**
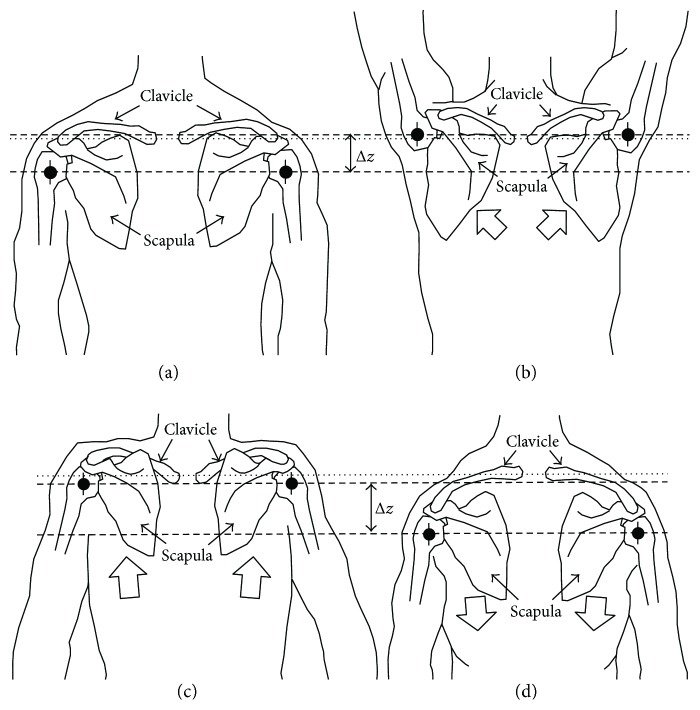
Coronal plane back view representing the CR displacement of the shoulder driven by the clavicle and scapula in (a) resting position, (b) abduction, (c) elevation, and (d) depression. Dotted lines represent the sternoclavicular joint position as reference and dashed lines represent the displacement limits of the acromioclavicular joint (∆*z*).

As shown in Figure 2, the shoulder (glenohumeral) joint CR depends on the clavicle and scapula interaction [28], which brings the widest shoulder displacement constraint. The scapula presents limited mobility in all its axes except for an angular displacement of 50°–60° about the axis normal to the ribcage (i.e., arm abduction) [29]. Moreover, muscles and ligaments next to the thorax limit scapular displacements and develop a lever that is driven by the sternoclavicular rotation and acromioclavicular elevation. The abovementioned interactions induce translational displacements of the shoulder CR, which occur during arm abduction in the first 20° of elevation [28]. Note that the arm abduction is highly dependent on the shoulder CR movements on the coronal plane, where the two extra DoF driven by the clavicle-scapula interaction extend the upper limb reachable workspace.

Arm motion analysis represents a topic of interest in upper limb exoskeleton design [11], particularly for rehabilitation devices. Such devices should optimally track patient movement with no undesired interaction forces, which results in a challenging task due to the mechanical constraints exerted by complex mechanisms found in the literature [10, 22]. This paper proposes a biomimetic kinematic model for upper limb exoskeletons, which aims to emulate the clavicle motion contribution to the shoulder complex in the coronal plane by adding an additional link. Also, a performance index is proposed that numerically evaluates the efficacy in which exoskeletons track the human arm mobility that is similar to indexes used to assess haptic interfaces [30].

The mobility of the shoulder complex depends on the tendons, ligaments, and muscles around and within each joint of the system. These interactions produce a highly variable angle range for every joint that limits arm mobility. The resulting reachable workspace emerges as an irregular shape, raising the challenge of properly fitting a robot workspace to the one generated by the human anatomy.

**Figure 3:**
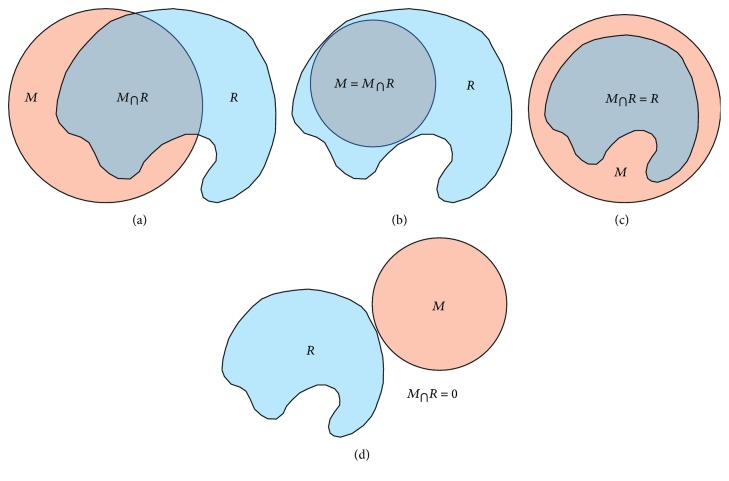
Fitting scenarios between human and exoskeleton workspaces: (a) partial intersection, (b) user workspace engulfs mechanism, (c) mechanism engulfs user workspace, and (d) no intersection.

Ideally, exoskeleton and human anatomical workspaces should fit perfectly. This does not occur, however, and therefore, it is important to assess the capability of an exoskeleton to emulate the human range of motion. For this paper, an approach that studies the abovementioned workspaces considers four cases: (i) when both workspaces partially intersect (Figure 3(a)), (ii) when the user workspaces engulf the mechanism (Figure 3(b)), (iii) when the mechanism workspace engulfs the user (Figure 3(c)), and (iv) when the workspaces do not intersect (Figure 3(d)). This paper proposes the anatomic fitting index *P*(*k*) to assess an exoskeleton potential to replicate human mobility.

(1) $$\begin{array}{c} P\left(k\right)=\left(1-k\right)\left(\frac{R\cap M}{R}\right)+k\left(\frac{R\cap M}{M}\right), \end{array}$$

where *M* and *R* represent the normalized exoskeleton and user workspaces, respectively, and *k* distributes the impact of each workspace usage index. An exoskeleton that perfectly emulates the human workspaces would have the maximum value possible *P*(*k*) = 1, while strongly unmatching workspaces would approach *P*(*k*) = 0. Moreover, *k* represents an index (between 0 and 1) that enables the determination of a *P*(*k*) dependent on the application of the modeled workspace usage. This flexibility given by the *k* factor enables asymmetrical weighing of the unmatching workspaces. For example, *k* = 1 would penalize heavily exoskeleton workspaces that overextend (see Figure 3(c)), while disregarding mechanisms that are unable to properly cover the arm workspace (see Figure 3(b)). Conversely, *k* = 0 would overlook the former and handicap the latter condition.

Section 3 will focus on the design of a novel upper limb exoskeleton, while the performance index presented in the previous paragraphs will be applied and discussed in Section 5.

## 3. Upper Limb Exoskeleton Design

The exoskeleton developed in this work presents natural shoulder abduction/adduction movements. This is possible by correctly considering and allocating the anatomical axes that drive those movements and simplifying components that do not contribute to said mobility. Namely, the shoulder joint (glenohumeral) presents a perceivable CR translation over the coronal anatomic plane (∆*z*, see Figure 2); while the acromioclavicular joint has a marginal contribution to the shoulder movement, and therefore, it is neglected in the design presented in the following paragraphs.

A deep analysis of the human upper limb anatomy begins with the study of shoulder mobility axes. The overlapping of the user and the exoskeletal system axes requires the allocation of the glenohumeral and sternoclavicular joint anatomic position in a virtual design environment. This work proposes a method to design an upper body exoskeleton based on 3D scanning vision technologies. The resulting 3D image of a subject forms an anthropomorphic surface from where the exoskeleton is designed. These systems' reliability is reported to be between 3 and 4 mm (structure sensor), while the minimum area of the regions of interest for the design process varies between 2 and 3 cm^2^. Notice that, since this method is based on anatomical position references, its effectiveness is affectedwhen used in subjects with anatomical abnormalities caused by trauma, genetics, or health conditions. The following paragraphs describe the scanning and design procedure in detail.

**Figure 4:**
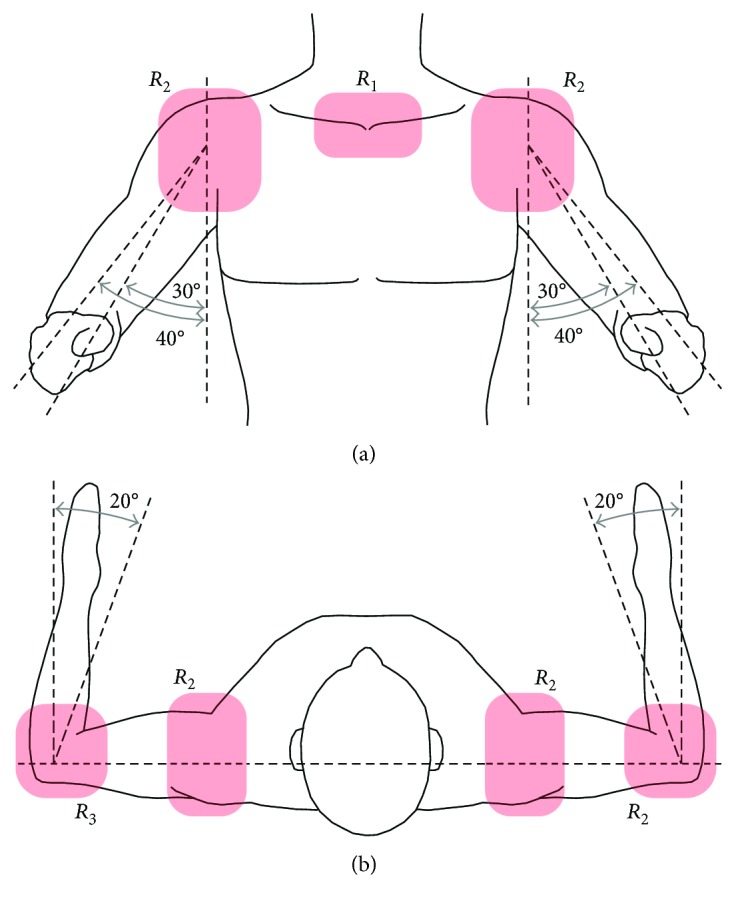
(a) Frontal view and (b) top view of the subject's scanning posture, where red-shaded areas are exposed.

**Figure 5:**
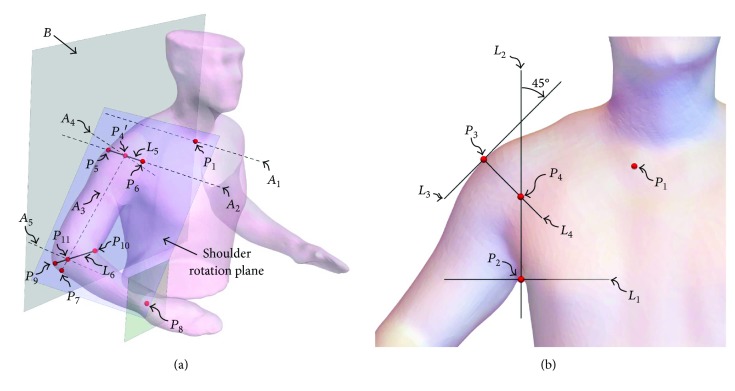
(a) Identification of the main rotational axes for the exoskeletal system and (b) first shoulder axis allocation from the subject coronal view. Plane *B* is positioned as a parallel reference to the coronal plane.

The scan begins by positioning the subject standing with a shoulder abduction of 30° to 40°, an elbow flexion of approximately 90°, and a shoulder internal rotation between 0° and 20°. This pose exposes the collarbone, shoulder, and elbow of the subject, while it improves the scan quality by avoiding any interference (see Figure 4). The obtained surface serves as a reference to allocate the main rotational axes of the upper limb mobility (axes *A*_1_ to *A*_5_, see Figure 5(a)), which can be determined with the following methods:

1. Allocate the *R*_1_ sternum area in the 3D workspace (see Figure 4(a)). Note that this surface encloses both the right and left sternoclavicular joints that appear as symmetrical bulges. Due to the reduced size of the bulge, consider that point *P*_1_ is concentrically placed at the sternoclavicular joint, where the rotational axis *A*_1_ is projected to intersect *P*_1_ and be normal to the coronal plane, as shown in Figure 5(a). Point *P*_1_ is found by rotating the 3D model and observing that its position on the bulge remains invariant after the rotation.
2. The shoulder adduction/abduction axis is denoted as *A*_2_ and is determined using the coronal plane view of the scanned surface (Figure 5(b)). From the surface contour projection to this plane, consider that point *P*_2_ is placed in the anterior axillary fold and is intersected by the horizontal line *L*_1_. Let *L*_2_ be perpendicular to *L*_1_ and intersect *P*_2_. Furthermore, consider *L*_3_ to be defined when *L*_2_ is rotated 45° clockwise and intersects *P*_3_ on the shoulder contour. Finally, the point of rotation *P*_4_ is located at the intersection of *L*_2_ and *L*_4_, which is perpendicular to *L*_3_ and intersects *P*_3_. Note that the rotational axis *A*_2_ is normal to the coronal plane and intersects *P*_4_ as presented in Figure 5(a).
3. The shoulder joint (glenohumeral) CR *P*_4_′ can be found when *P*_4_ is translated along the *A*_2_ axis. Consider that *P*_5_ and *P*_6_ represent points on the surface of the deltoids muscle that are intersected by *A*_2_. The center of rotation *P*_4_′ is placed at the middle of *L*_5_, which is defined by points *P*_5_ and *P*_6_. The shoulder rotational axis *A*_3_ is thus denoted by the intersection of *P*_4_′ and *P*_7_, which is located on the elbow at the olecranon base (see Figure 5(a)). Note that given the reduced size of the ulna, *P*_7_ is determined using a similar approach to *P*_1_ as described in (1).
4. The *A*_4_ axis that describes the shoulder flexion/extension is defined in Figure 5(a) to be orthogonal to axes *A*_2_ and *A*_3_, intersecting *P*_4_′.
5. Let the shoulder rotation plane be defined by the axis *A*_3_ and point *P*_8_, which is located in the center of the wrist cross section. Consider *P*_9_ and *P*_10_ on the abovementioned plane to be the inner and outer corners of the arm folding at the elbow joint, respectively, which define *L*_6_. The *A*_5_ axis is normal to the shoulder rotational plane and intersects point *P*_11_, which at the same time intersects *A*_3_ and *L*_6_ (Figure 5(a)).

**Figure 6:**
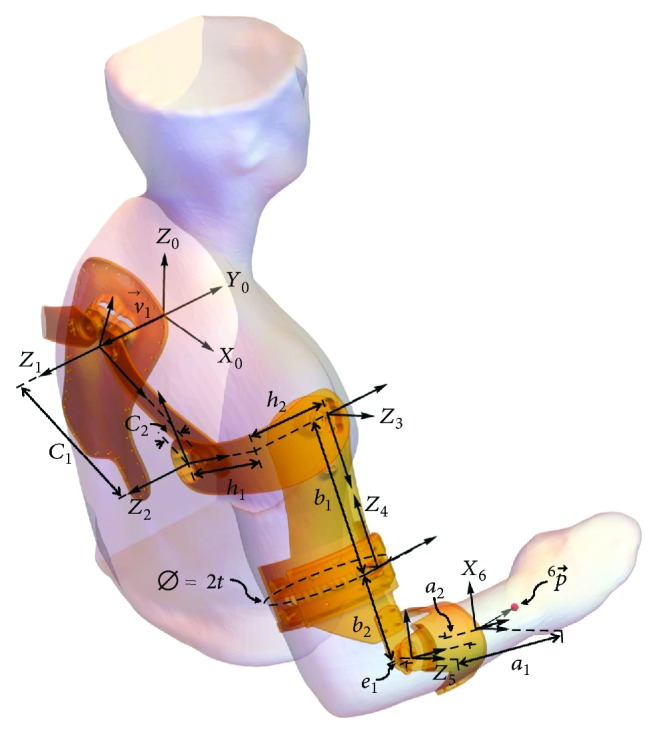
Exoskeleton mechanism design with aligned axes.

Note that axes *A*_1_ to *A*_5_ represent the position of the mechanical joints, which are interconnected by links that compose the exoskeleton kinematic chain. In order to avoid self-constraining from joints, axis *A*_3_ is constructed as a circular sliding radial joint (with a 2*t* diameter) that is placed at the middle of the arm and works in addition as an effective strap for the arm (Figure 6). Furthermore, the links can be designed as offsets of the 3D image, resulting in a significantly ergonomic physical interface.

Figure 6 presents the exoskeletal mechanism that includes the local reference frames used in the forward kinematic analysis. The construction of this model was performed by using 3D printing, which allows to rapidly test every part of the system for analysis purposes. Additionally, Table 1 shows the Euler parameters (*zxz*) for the kinematic representation of the mechanism, where every row represents an homogeneous transform matrix **E**_*i*_ that is defined as

(2) $$\begin{array}{c} E_{i}=\left[\begin{array}{cc} R_{i}{\left(\alpha ,\beta ,\gamma \right)}_{3\times 3} & {\vec{v}}_{i}\\ {\vec{0}}^{T} & 1 \end{array}\right], \end{array}$$

where **R**_*i*_(*α*, *β*, *γ*) is the 3 × 3 rotation matrix of the local frame *i*, ${\vec{v}}_{i}$ is the position vector of the local reference frame *i*, and $\vec{0}$ is the 3 × 1 zero vector.

The homogeneous transformation **M**_*T*_ that expresses the exoskeleton end effector is obtained by premultiplying the homogeneous transformation matrices **E**_*i*_ for *i* = 1 to *i* = 6 as

(3) $$\begin{array}{c} M_{T}=E_{1}\cdot E_{2}\cdot \cdots \cdot E_{6}. \end{array}$$

Hence, the global position vector ${}^{0}\vec{P}$ of a known point in the last local reference frame ${}^{6}\vec{P}$ is obtained as

(4) $$\begin{array}{c} {}^{0}\vec{P}=M_{T}\cdot^{6}\vec{P}. \end{array}$$

Let ${}^{6}\vec{P}$ be the position of a point at the middle of the forearm cross section at an *m* distance from the *A*_5_ axis, where

(5) $$\begin{array}{c} {}^{6}{\vec{P}}^{T}=\left[0\;a_{2}-e_{1}-t\;m-a_{1}\;1\right], \end{array}$$

and substituting (5) in (4) with the dimensional parameters listed in Table 1, the position variables of ${}^{0}\vec{P}$ result as follows:

(6) $$\begin{array}{c} {}^{0}{\vec{P}}_{x}=s\left(\gamma_{1}+\gamma_{2}\right)\left(-s\left(\gamma_{3}\right)c\left(\gamma_{4}\right)s\left(\gamma_{5}\right)m+c\left(\gamma_{3}\right)c\left(\gamma_{5}\right)m+c\left(\gamma_{3}\right)\left(b_{1}+b_{2}\right)\right)-c\left(\gamma_{1}+\gamma_{2}\right)s\left(\gamma_{4}\right)s\left(\gamma_{5}\right)m+c\left(\gamma_{1}\right)c_{1}+x_{1}, \end{array}$$

(7) $$\begin{array}{c} {}^{0}{\vec{P}}_{y}=\left(c\left(\gamma_{3}\right)c\left(\gamma_{4}\right)s\left(\gamma_{5}\right)+s\left(\gamma_{3}\right)c\left(\gamma_{5}\right)\right)m+s\left(\gamma_{3}\right)\left(b_{1}+b_{2}\right)+h_{2}-c_{2}+y_{1}, \end{array}$$

(8) $$\begin{array}{c} {}^{0}{\vec{P}}_{z}=-c\left(\gamma_{1}+\gamma_{2}\right)\left(-s\left(\gamma_{3}\right)c\left(\gamma_{4}\right)s\left(\gamma_{5}\right)m+c\left(\gamma_{3}\right)c\left(\gamma_{5}\right)m+c\left(\gamma_{3}\right)\left(b_{1}+b_{2}\right)\right)-s\left(\gamma_{1}+\gamma_{2}\right)s\left(\gamma_{4}\right)s\left(\gamma_{5}\right)m+s\left(\gamma_{1}\right)c_{1}+z_{1}. \end{array}$$

The mathematical model in (6), (7), and (8) serves as the main tool for the further analysis of the designed exoskeletal mechanism. Also, the forward kinematic construction from Table 1 and (6), (7), and (8) considers anatomic variability between subjects in their parameters such as clavicle length (*c*_1_), arm length (*b*_1_ + *b*_2_), and arm thickness (2*t*); these brings to the exoskeleton a high adaptability to differently shaped users, easing its construction and modelling. A numerical example of the abovementioned model is presented in order to validate its plausibility and will be further used to obtain the analytical workspace to assess the mechanism performance.

### 3.1. Numerical Example

The analytical results of the mathematical model are validated in this paper by comparing a numerical example with a computer simulation. The simulation is performed by using a CAD environment to analyze the trajectory of a point on the exoskeleton mechanism. During the simulation, the mechanism is subjected to controlled angular displacements about the rotational axes from a given initial position, beginning at time *t* = 0 s with a starting angular position of 0° for axes *A*_1_ to *A*_4_ and 90° for axis *A*_5_, while the ending angular position at time *t* = 10 s is 15° for joints 1 to 4 and 105° for joint 5. This angular position change is applied at a constant rate for every joint in both CAD simulation and the numerical example.

Furthermore, the dimensional parameters listed in Table 1 are obtained from the CAD model and presented in Table 2. Note that Table 2 only contains the necessary parameters for the correct computation of (6), (7), and (8). The mean absolute error of the resulting trajectories is 3.71292 × 10^−9^ mm, which can be attributed to the intrinsic measurement error of the CAD environment.

## 4. Experimental Setup with Motion Capture System

Section 2 proposed the determination of a performance index for mechanisms intended to act as exoskeletal systems, where the fitting between workspaces plays a crucial factor in the interaction between users and devices. This section presents the experimental setup used to obtain the workspace volumes for the human arm (anatomical) and the exoskeleton mechanical model (analytical) based on their reachable positions. In order to have comparable data, both volumes were normalized to a reference frame and scaled to equivalent reference measurements. Normalization is possible by using a vision system as a tool for obtaining the anatomical workspace data, which allocates a reference frame that can be later shared with the analytical workspace. Also, the mathematical model presented in Section 3 ((6), (7), and (8)) allows the analytical construction of a variety of exoskeletal mechanisms, where dimensional parameters can freely adjust according to the anatomical measurements of the final exoskeleton user, enabling scalability between workspaces.

The VICON tracking system captures the motion of retroreflective markers with the use of a set of infrared sensitive cameras fixed in a sunlight-free room. These cameras, which have an infrared stroboscopic ring lamp around their lenses, detect the light that is reflected on spherical markers that are strategically positioned over the tracked body. VICON has a location system that creates a static workspace by using a set of cameras based on homography in which a marker can be detected by a triangulation of at least two cameras. This system is able to allocate a marker in the 3D workspace for every observed frame. Through the NEXUS software, VICON users are able to create complex models of marker configurations, establish relationships amongst them, and label markers to obtain individual series of position, velocity, and acceleration data.

The experiments presented in this paper produce a 3D surface corresponding to the reachable workspace for an asymptomatic subject arm. Using the VICON tracking system, data series of position can be retrieved by recording trajectories of markers allocated over the subject. These trajectories cover every reachable arm position in order to create a complete cloud of points that are further converted into a surface.

**Figure 7:**
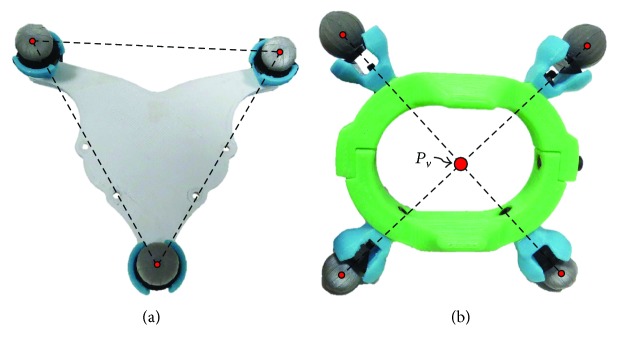
(a) Back marker plate and (b) bracelet marker plate.

Placing markers directly over a subject body (skin or clothes) produce positioning errors due to the common uncertainties given by the relative motion between the markers and the user or its interface. A solution to this problem is to place the markers over rigid bodies worn by the subject [18]. Two marker plates are designed to act as rigid bodies in order to ease the subsequent data processing: (i) the static reference frame and (ii) the reference for the trajectories creation. Both plates are manufactured using 3D printing technologies. The static reference is further aligned with VICON's global reference frame. This requires at least three points to establish its position and orientation; thus, this marker plate is designed as an equilateral triangle (see Figure 7(a)). Consider that the marker plate for the static reference frame is fixed at the back of the subject and coincident with the first reference frame of the exoskeleton kinematic chain (Figure 6) for the convenience of the analysis. Also, this marker plate is attached to the subject by contact at the upper and lower back and by the use of an orthopedic belt/posture corrector, which is designed to freely allow shoulder mobility (including scapula and clavicle).

The second marker plate that is used to detect the subject movements is located on the forearm in order to include the shoulder complex and the elbow joint motions. For this purpose, a wrist bracelet is designed with four markers allocated as corners of a square (Figure 7(b)), allowing the vision system to identify a minimum of two markers for every recorded frame. Note that this design allows the further creation of a virtual point at the middle of the wrist, which is later used to create the needed trajectories. The virtual point *P*_*v*_ is created by finding the average position between the middle points of the diagonals created by the markers. Although the middle points of the diagonals should be equal in position, this method minimizes the intrinsic error that the vision system carries when locating each marker in space. Note that this virtual point corresponds to ${}^{6}\vec{P}$ as presented in Figure 6.

**Figure 8:**
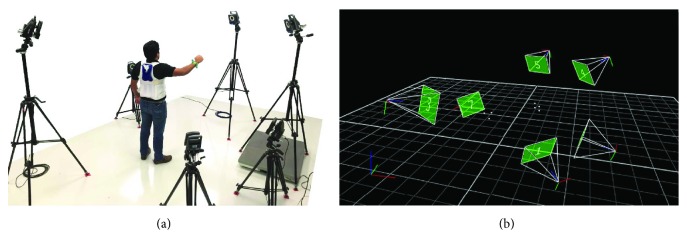
VICON setup. (a) Set of cameras and marker plates as positioned for the recording sessions. (b) VICON digital environment NEXUS, where every nonmarker is deleted from the digital scenario.

Once the reference frames are manufactured and allocated over an asymptomatic subject, recordings of its arm mobility are taken. The best data quality is achieved by calibrating the VICON system to enclose a static workspace of approximately 1.5 meters width, length, and height. This configuration shows a proper noise reduction, leading to an overall accuracy increase. Moreover, the marker plate use allows to assess data reliability, typically showing accumulated squared errors between 3 and 6 mm^2^ for every marker relative position as shown in previous work [18]. Figures 8(a) and 8(b) show the VICON cameras setup and its virtual representation in NEXUS, respectively. Several recordings (11) of pseudorandom movements are taken after calibration at 100 fps. The goal of the recording session is to cover every reachable region by the subject, which will result in the determination of complete workspace surfaces when all subject sessions are merged in a single data set.

The resulting position data yields the exoskeleton reachable workspace. Similarly, the exoskeleton kinematic chain produces the system mathematical model (see Table 1). This model receives the angular displacement about each axis (*A_i_*) at a given pose and returns the position of a point in the last reference frame of the kinematic chain.

**Figure 9:**
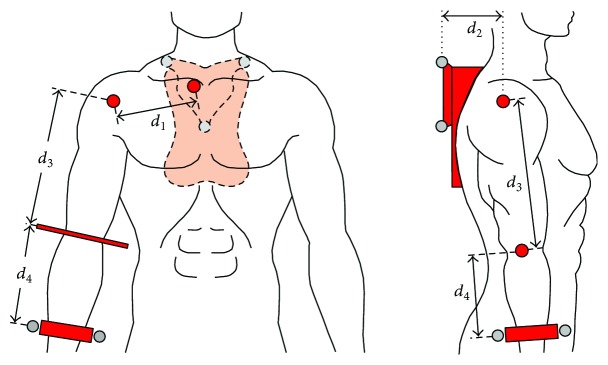
Anatomic dimensional parameters of the test according to Tables 1 and 3. Light gray circles represent the corresponding VICON markers.

To obtain a mathematical model for a particular user and for the purpose of further comparison, dimensional parameters are taken from the same subject from whose the anatomical workspace is later obtained. These parameters (*d*_*j*_) correspond to those in (6), (7), and (8) as elements of the *v*_*i*_ position vectors and are directly related to particular anatomical measurements as shown in Figure 9 and Table 3. These parameters are obtained by measuring the anatomical distances between sternoclavicular-glenohumeral and glenohumeral elbow joints (*d*_1_ and *d*_3_, resp.), the horizontal distance between the back marker plate and the glenohumeral joint over the sagittal plane (*d*_2_), and the distance between the elbow joint and the position of the wrist marker plate over the subject's forearm (*d*_4_).

Finally, it is necessary to provide an input series for the model in order to obtain a workspace point cloud, where each input data consists of an angular displacement per joint. These sets are generated by sweeping each joint throughout its range of motion (see Table 4). The resulting point cloud can be later converted into a reachable surface and then into a workspace volume.

Upper limb exoskeleton designs present three DoF in related work found in the literature [1, 16, 25, 26]. This paper proposes one additional DoF to the sternoclavicular joint rotation over the anatomical coronal plane (see Figures 1 and 2) and tests its performance with the abovementioned index. The following section tests this design with two particular models for comparison: (i) a design with no clavicle rotation that emulates the common exoskeleton design for upper limbs, whose position vector ${}^{0}\vec{O}$ to the end-effector point results as

(9) $$\begin{array}{c} {}^{0}\vec{O}{=}^{0}\vec{P}\left|{}_{\gamma_{1}=0}\right., \end{array}$$

where ${}^{0}\vec{P}$ is obtained from (4) and represents the fixation of the axis *A*_1_ (see Figure 5(a)); and (ii) a model where the clavicle axis *A*_1_ (variable *γ*_1_) rotates over the transversal plane (parallel to the *z*_0_ axis, Figure 6), whose position vector ${}^{0}\vec{Q}$ is obtained from a variation of 4 as follows:

(10) $$\begin{array}{c} {}^{0}\vec{Q}=N_{T}\cdot^{6}\vec{P}, \end{array}$$

where **N**_*T*_ results from

(11) $$\begin{array}{c} N_{T}=E_{7}\cdot E_{8}\cdot E_{3}\cdot E_{4}\cdot E_{5}\cdot E_{6} \end{array}$$

and is obtained by constructing the homogeneous matrices **E**_7_ and **E**_8_ with the Euler parameters in Table 5 and constructing **E**_3_ to **E**_6_ with the respective parameters in Table 1. All three models share the input parameters defined in Table 4.

## 5. Experimental Results

The anatomic fitting index that is proposed in this work to compare the exoskeleton systems and human reachable workspace is presented in Section 2. Equation (1) proposed the performance index *P*(*k*) depending on the volume of the human workspace, the exoskeleton workspace, the intersection of both volumes, and the index *k*.

Section 4 presented the methods to obtain the 3D coordinates of the anatomical workspace with the VICON vision system and the 3D coordinates of the analytical workspace with the exoskeleton mathematical model presented in Section 3 ((4)). Moreover, Section 4 established two particular variations of the said model for clavicle motion comparison purposes, which are obtained from (9) and (10). The 3D coordinates from the 3 analytical models and the anatomical data are processed to obtain and compare their workspaces using the performance index *P*(*k*) (1).

The list of 3D points for each experiment was processed using the concave hull algorithm [31] in the commercial software Wolfram Mathematica. The Delaunay algorithm found in the TetGen library was used to obtain the first surface approximation, which contains circumspheres with a set of 4 points from the data that become the vertices of a tetrahedron. A convex hull mesh was formed using all these tetrahedra.

**Figure 10:**
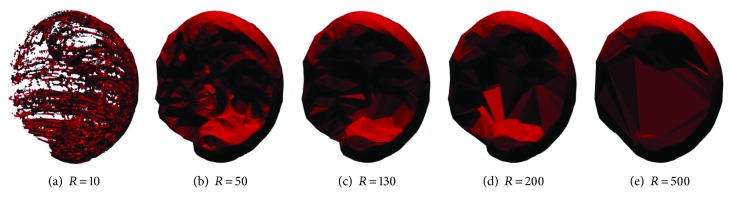
Workspace for different radius *R*.

A refined approximation of the workspace was achieved by converting the convex hull mesh into a concave hull mesh, which was obtained comparing the radius of the circumspheres *r*_*i*_ for all the obtained tetrahedra to a maximum permitted radius *R* and neglecting all the point sets of the tetrahedra whose radius *r*_*i*_ > *R*. The criterion for setting the parameter *R* was determined by identifying the minimum value of *R* that will avoid the occurrence of holes on the surface of the model.  Figure 10 presents the workspace obtained for different radius *R*, where (a) and (b) presented holes in the surface, (d) and (e) lost details in the concave zone, and (c) represented an acceptable balance between surface quality and concave details.

The generated data represent clusters of tetrahedra that approximate the shape of each point cloud that eliminates all the internal triangular faces to generate a single surface. Every 4-point set can be subdivided into 4 subsets of 3 points representing the 3 vertexes on each of the 4 tetrahedron faces, where all the internal faces are shared between 2 tetrahedra. Thus, a comparison of all the subsets enables the removal of all the internal faces leaving the external triangular faces data of the concave hull mesh.

**Figure 11:**
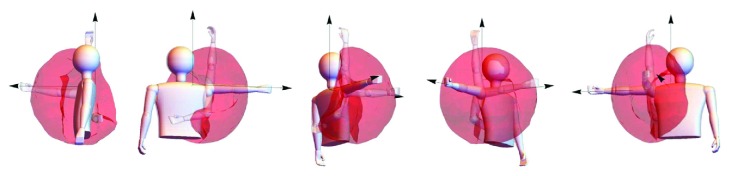
Experimental human workspace for the upper limb.

Finally, the 3D model estimation from the data was exported to an STL format file to obtain the volume of the workspace.  Figure 11 presents 5 equally spaced views of the 3D model generated from the anatomical workspace experiments.

**Figure 12:**
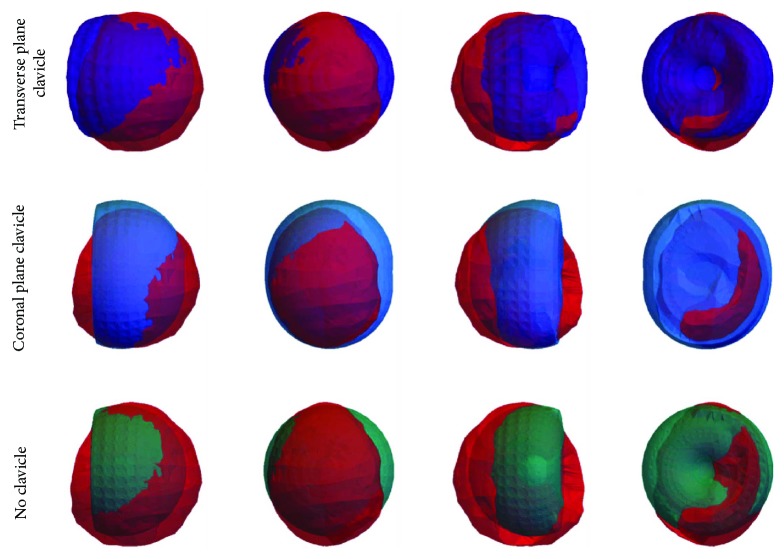
Intersections between real and modeled workspaces.

The volume of the 4 workspaces (1 anatomical and 3 analytical) is summarized in Table 6. Also, the intersection between the analytical and anatomical volumes is shown in Figure 12 and quantified in Table 7.

**Figure 13:**
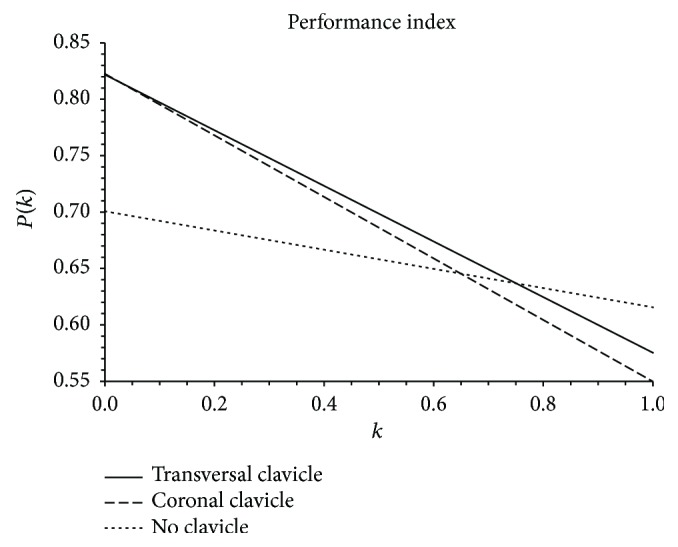
Performance index plot for every *k*.

The intersecting volume data leads to the determination of the anatomic fitting index *P*(*k*) using (1). In addition, Table 7 shows both extremes of the *P*(*k*) index, which provide insight of the user and mechanism effective workspace when *k* = 0 and *k* = 1, respectively. These extremes of the index can also be used to make a thorough analysis of the fitting performance by plotting *P*(*k*) for every possible *k*, as seen in Figure 13. It can be observed that a higher line midpoint represents a better correspondence between the analytical and anatomical workspaces, while a lower line midpoint is a result of a poor fitting. Also, the slope of the line represents the contrast between workspace fittings, where ideal equal fittings result in a slope of 0. Positive or negative slopes represent a higher proportional fitting of the analytical or anatomical workspace, respectively. Note that these parameters, obtained from *P*(*k*), can take absolute values between 0 and 1, offering additional comparison data between analytical models.

From Figure 13, it can be observed that the clavicle models cover more of the human achievable workspace (*P*(0) = 0.82), but they also allow positions that are nonreachable by the human, having a *P*(1) = 0.57 for the transverse plane clavicle and *P*(1) = 0.55 for the coronal plane clavicle. The model with no clavicle covers less of the human workspace volume (*P*(0) = 0.7) but allows less nonreachable user positions (*P*(1) = 0.61). At 0.64 *<* *k* *<* 0.75, the two clavicle models present similar performance to the one without clavicle (0.637 *<* *P*(*k*) *<* 0.645). From these data, it can be implied that in general, the exoskeletons with clavicle structures present a better performance that the model lacks clavicle linkages. Also, the modified model with transversal clavicle rotation shows a higher *P*(1), which suggests that a further analysis of a model with enhanced mobility at the clavicle over both planes (coronal and transversal) is required. Lastly, for *k* *>* 0.64, the performance index of the exoskeleton with no clavicle outperforms the indexes of the exoskeletons with no clavicle, meaning that there is a lower nonreachable workspace than the clavicle variants.

## 6. Conclusions

This paper presents a method to evaluate exoskeleton performance by means of reachable workspace comparison, where the proposed anatomic fitting index assess the compatibility of the exoskeleton and the human biomechanics of different gross motion tasks. This way, high performance enables to widen exoskeleton applications, such as in rehabilitation tasks, by emulating the shoulder CR drifting characteristic, which improves the range of motion of the user through the application of clavicle exoskeletal mechanisms.

This paper proposes an architecture for upper limb exoskeletons that considers a mobile CR of the shoulder while easily adaptable to different user shapes due to its model construction. Also, its drifting CR characteristic is given by the inclusion of a biomimetic link that emulates the sternoclavicular joint in the coronal plane. The variant position of the shoulder CR plays an important role in the arm motion, where its wider translation can be seen from the first 20° abduction and is primarily performed by the clavicle. The said architecture is also analyzed with forward kinematics to obtain a mathematical model of its end-effector position, which is also validated with a CAD-based simulation.

3D scanning vision technologies ease the exoskeleton design process. Together with the proposed design method, it is possible to allocate the rotational axes for a variety of subjects. The use of an anthropomorphic surface in a digital environment improves the biomimetic design by properly placing axes and developing ergonomic linkages. Also, motion tracking systems allow the obtention of anatomic workspaces to be used for comparison and biomimetic analysis purposes.

The proposed design shows a 17.1% increase of the anatomic workspace usage by the inclusion of the clavicle contribution to the coronal plane when compared to nonclavicle designs, while it only expands the nonreachable workspace of the user by 6%. Furthermore, an assessment of a modified transversal clavicle model shows an equally enhanced performance.

Nonreachable volumes are the result of the differences between the mechanical and anatomical joints. While anatomical joints have posture-related constraints given by the muscles and tendons, mechanical joints have constant complete mobility, which results in an important change of shape in the workspace surfaces. Also, the proposed mechanism aims to evaluate its complete motion; hence, the constraining volumes (subject body) are not considered in the reachable surface acquisition. Furthermore, most of the unmatching volumes can be eliminated by control, where constraining actuators can actively apply virtual barriers to the mechanical system.

Future work will focus on the assessment of a variety of state-of-the-art exoskeleton devices to evaluate their performance, bringing a wider insight of the possible applications of these mechanisms. Also, motion tracking systems will be further used to allocate the rotational axes of the anatomic system and to develop a deep analysis of the asymptomatic upper limb biomechanics.

## Figures and Tables

**Table 1:** Euler parameters (*zxz*) for the kinematic model of the proposed exoskeletal system.

				*v*_*i*_		
*i*	*α*	*β*	*γ*	*d*_*x*_	*d*_*y*_	*d*_*z*_

1	0	*π /*2	*γ*_1_	*x*_1_	*y*_1_	*z*_1_
2	0	0	*γ*_2_	*c*_1_	0	*c*_2_
3	−*π/*2	−*π/*2	*γ*_3_	*h*_1_	0	−*h*_2_
4	*π /*2	−*π/*2	*γ*_4_	*b*_1_	*t*sin(*γ*_4_)	*t*cos(*γ*_4_) − *h*_1_
5	0	*π /*2	*γ*_5_ − *π/*2	0	−*e*_1_	−*b*_2_
6	*π /*2	*π /*2	0	*a*_1_	0	−*a*_2_

**Table 2:** Dimensional parameters obtained from the CAD model.

Parameter	*x*_1_	*y*_1_	*z*_1_	*c*_1_	*c*_2_	*h*_2_	*b*_1_	*b*_2_	*m*
Value (mm)	0	−14	0	172.55	21.89	101.23	161.29	91.79	200

**Table 3:** Model parameters obtained from the subject under analysis.

Anatomical measurement	*d*_1_	*d*_2_	*d*_3_	*d*_4_
Equation equivalent	*c*_1_	*h*_2_ − *c*_2_ + *y*_1_	*b*_1_ + *b*_2_	*m*

**Table 4:** Angle set ranges.

Joint	Min	Max
Sternoclavicular	−5	15
Shoulder 1	−40	160
Shoulder 2	−60	180
Shoulder 3	−100	20
Elbow	0	160

**Table 5:** Euler parameters (*zxz*) for the transversal clavicle variant of the proposed exoskeletal system.

				*v*_*i*_		
*i*	*α*	*β*	*γ*	*d*_*x*_	*d*_*y*_	*d*_*z*_

7	0	0	*γ*_1_	*x*_1_	*y*_1_	*z*_1_
8	0	*π /*2	*γ*_2_	*c*_1_	−*c*_2_	0

**Table 6:** Raw volume data.

Workspace	Volume (m^3^)
Human arm	0.2756
No clavicle exoskeleton	0.3137
Coronal clavicle exoskeleton	0.4123
Transverse clavicle exoskeleton	0.3936

**Table 7:** Workspace intersections.

Workspace intersection with the human arm	Volume (m^3^)	*P*(0)	*P*(1)
No clavicle exoskeleton	0.1931	0.7	0.61
Coronal clavicle exoskeleton	0.2267	0.82	0.55
Transverse clavicle exoskeleton	0.2264	0.82	0.57
